# Pembrolizumab provides long‐term survival benefits in advanced non‐small cell lung cancer: The 5‐year outcomes of the KEYNOTE‐024 trial

**DOI:** 10.1111/1759-7714.14193

**Published:** 2021-10-13

**Authors:** Fengtan Li, Xifeng Dong

**Affiliations:** ^1^ Department of Radiology Tianjin Medical University General Hospital Tianjin China; ^2^ Department of Hematology Tianjin Medical University General Hospital Tianjin China

Lung cancer is one of the most frequently diagnosed cancers in the world with more than 2 million new cases diagnosed every year.^1^ As the largest developing country worldwide, China has more lung cancer diagnoses and fatalities than any other countries.^2^, ^3^ Non‐small cell lung cancer (NSCLC) is the most common type of lung cancer comprising up to 85% of lung cancer cases. Platinum‐based chemotherapy is a traditional treatment for patients with advanced NSCLC. However, even with platinum‐based chemotherapy, the 5‐year overall survival (OS) rate of these patients is still less than 7%.^4^, ^5^ In the 2010s, immunotherapy, especially treatment with pembrolizumab (a monoclonal antibody of programmed cell death protein 1 [PD‐1] antibody), altered the treatment landscape for NSCLC patients without driver mutations.^6^, ^7^ Previous clinical trials have shown that pembrolizumab monotherapy more effectively improved the OS of advanced NSCLC patients without programmed death‐ligand 1 (PD‐L1) expression and oncogenic epidermal growth factor receptor (*EGFR*), or anaplastic lymphoma kinase (*ALK*) mutations compared with platinum‐based chemotherapy.^8^, ^9^ Additionally, pembrolizumab in combination with chemotherapy has been shown to improve OS versus chemotherapy alone for advanced NSCLC patients without driver mutations, regardless of PD‐L1 expression.^10^, ^11^

The first randomized, phase III study comparing the efficacy of pembrolizumab monotherapy and platinum‐based chemotherapy is KEYNOTE‐024. This study enrolled patients with previously untreated NSCLC who had a PD‐L1 tumor proportion score (TPS) ≥ 50%. It demonstrated that pembrolizumab monotherapy was superior to platinum‐based chemotherapy in the first‐line setting. The second interim analysis of KEYNOTE‐024 showed that progression‐free survival (PFS) and OS of the pembrolizumab arm were significantly improved as compared with those of the chemotherapy arm (PFS: hazard ratio [HR] = 0.50, 95% confidence interval [CI]:  0.37–0.68, *p* < 0.001; OS: HR = 0.60, 95% CI: 0.41–0.89, *p* = 0.005).^12^ The subsequent analysis of KEYNOTE‐024 demonstrated that OS continued to be improved for pembrolizumab monotherapy versus platinum‐based chemotherapy (HR = 0.63, 95% CI:  0.47–0.86).^8^

For NSCLC, if there is no recurrence within 5 years after initial diagnosis, the chance of recurrence later is very small. Therefore, 5‐year survival is considered to be an important milestone in the treatment of NSCLC. However, since few patients with advanced NSCLC are alive 5 years after initial diagnosis, the 5‐year survival data of these patients are rarely reported. Only one study (KEYNOTE‐001; a single‐arm, phase Ib trial) previously reported that the 5‐year OS rate of advanced NSCLC patients treated with pembrolizumab monotherapy was 29.6% (95% CI: 7.7%–56.1%).^13^

Recently, the 5‐year safety and efficacy outcomes of KEYNOTE‐024 were published in the *Journal of Clinical Oncology* by Reck et al.^14^ In KEYNOTE‐024, 305 NSCLC patients (previously untreated, PD‐L1 TPS ≥50%, without sensitizing *EGFR* or *ALK* mutations) were randomly assigned (1:1) to pembrolizumab or platinum‐based chemotherapy. The patients assigned to a pembrolizumab arm were treated with 200 mg pembrolizumab once every 3 weeks for up to 35 cycles. The patients who received platinum‐based chemotherapy could cross over to pembrolizumab once the disease had progressed. The median time from randomization to data cutoff in this study was 59.9 months (range: 55.1–68.4 months). There were 65.6% (99/151) of patients in the chemotherapy arm subsequently treated with anti‐PD‐1 or anti‐PD‐L1 therapy. The authors found that the median OS of the pembrolizumab group (26.3 months, 95% CI: 18.3–40.4 months) was significantly longer than that of the chemotherapy group (13.4 months, 95% CI: 9.4–18.3 months). Similarly, Kaplan–Meier analysis showed that the 5‐year OS rate of the pembrolizumab group was significantly better than that of the chemotherapy group (31.9% vs. 16.3%). Among the 39 patients who received 35 cycles of pembrolizumab monotherapy, 32 (82.1%) patients were still alive at the time of the data cutoff (about 5 years from the initial treatment). The treatment‐related advanced event incidence of pembrolizumab was lower than that of platinum‐based chemotherapy, indicating that pembrolizumab was well tolerated. Furthermore, the safety of pembrolizumab monotherapy did not decrease with the prolonged treatment exposure.

The data from KEYNOTE‐024 indicated that pembrolizumab monotherapy could provide meaningful improved patient outcomes over platinum‐based chemotherapy for advanced NSCLC patients with PD‐L1 TPS ≥ 50% but without sensitizing *EGFR* or *ALK* mutations in the first‐line treatment setting. The median OS in the pembrolizumab group was at least 1 year longer than that in the chemotherapy group, and the 5‐year OS rate of the pembrolizumab group was almost twice that of the chemotherapy group. These data further confirm the 5‐year OS outcomes in the KEYNOTE‐001 trail. It is worth noting that most of the patients in the chemotherapy group received pembrolizumab treatment after experiencing disease progression. This high effective crossover rate may have reduced the observed treatment effect for pembrolizumab monotherapy versus platinum‐based chemotherapy. However, the outcomes between the two groups still showed huge differences, which further illustrated that the treatment effect of pembrolizumab monotherapy was very significant. In addition, the vast majority of patients who completed 35 cycles of pembrolizumab monotherapy were still alive at data cutoff, and nearly half of them survived without progressive disease or subsequent treatment, indicating that the treatment duration of pembrolizumab continued for at least 2 years. Moreover, of the 12 patients who received a second course of pembrolizumab due to subsequent progression, eight patients were still alive at data cutoff, and five survived without progressive disease, which indicated that pembrolizumab retreatment upon progressive disease is feasible and effective.

Other clinical trials demonstrated the benefit of pembrolizumab for other NSCLC patient populations. For example, the KEYNOTE‐042 study^9^ showed that pembrolizumab could improve OS in advanced NSCLC patients with PD‐L1 TPS >1% and without oncogenic *EGFR* or *ALK* mutations as compared with chemotherapy. Furthermore, the KEYNOTE‐189^10^, ^11^ and KEYNOTE‐407^15^ study demonstrated that pembrolizumab in combination with chemotherapy could improve OS versus placebo in combination with chemotherapy in nonsquamous and squamous advanced NSCLC without *EGFR* or *ALK* mutations and regardless of PD‐L1 expression, respectively. Trend analysis of lung cancer epidemiological data may partly reflect the impact of new therapies on disease state of lung cancer. Howlader et al.^16^ reported that incidence‐based mortality for both sexes between 2006 and 2016 declined at a rate of 2.3% to 6.3% per year. They believed that the emergence of new therapies to treat lung cancer has led to the continuous decline in lung cancer mortality. The 5‐year results of KEYNOTE‐024 indicate that pembrolizumab monotherapy may further reduce lung cancer mortality at the population level.

Overall, the 5‐year outcomes of the KEYNOTE‐024 study indicated that pembrolizumab, as the first‐line treatment of advanced NSCLC with a PD‐L1 TPS ≥50%, provides a clinically meaningful long‐term OS benefits versus platinum‐based chemotherapy. Pembrolizumab is expected to transform advanced lung cancer into a treatable chronic disease.

## CONFLICT OF INTEREST

The authors declare no competing interests.
